# Glycolysis Induces MCJ Expression That Links T Cell Proliferation With Caspase-3 Activity and Death

**DOI:** 10.3389/fcell.2019.00028

**Published:** 2019-03-11

**Authors:** Michael A. Secinaro, Karen A. Fortner, Cheryl Collins, Mercedes Rincón, Ralph C. Budd

**Affiliations:** Vermont Center for Immunology and Infectious Diseases, Larner College of Medicine, University of Vermont, Burlington, VT, United States

**Keywords:** caspase-3, MCJ, glycolysis, T cells, cell death

## Abstract

An effective adaptive immune response requires rapid T cell proliferation, followed by equally robust cell death. These two processes are coordinately regulated to allow sufficient magnitude of response followed by its rapid resolution, while also providing the maintenance of T cell memory. Both aspects of this T cell response are characterized by profound changes in metabolism; glycolysis drives proliferation whereas oxidative phosphorylation supports the survival of memory T cells. While much is known about the separate aspects of T cell expansion and contraction, considerably less is understood regarding how these processes might be connected. We report a link between the induction of glycolysis in CD8^+^ T cells and upregulation of the inhibitor of complex I and oxidative phosphorylation, methylation-controlled J protein (MCJ). MCJ acts synergistically with glycolysis to promote caspase-3 activity. Effector CD8^+^ T cells from MCJ-deficient mice manifest reduced glycolysis and considerably less active caspase-3 compared to wild-type cells. Consistent with these observations, in non-glycolytic CD8^+^ T cells cultured in the presence of IL-15, MCJ expression is repressed by methylation, which parallels their reduced active caspase-3 and increased survival compared to glycolytic IL-2-cultured T cells. Elevated levels of MCJ are also observed *in vivo* in the highly proliferative and glycolytic subset of CD4^-^CD8^-^ T cells in Fas-deficient *lpr* mice. This subset also manifests elevated levels of activated caspase-3 and rapid cell death. Collectively, these data demonstrate tight linkage of glycolysis, MCJ expression, and active caspase-3 that serves to prevent the accumulation and promote the timely death of highly proliferative CD8^+^ T cells.

## Introduction

The adaptive immune response is characterized by rapid proliferation of responding T cells followed by equally rapid cell death. These two processes need to be tightly coordinated, lest this result in excessive expansion or loss of T cells. Whereas much is known about the metabolic shifts leading to T cell proliferation and the death pathways resulting in contraction, less is appreciated about the possible close linkage of these two processes. Understanding this connection is critical to the design of T cell immunotherapy where enormous expansion of T cells is performed *ex vivo* using exogenous cytokines followed by the need for the cells to survive *in vivo* when infused in patients (Hollyman et al., 2009; Tumaini et al., 2013; Geyer et al., 2018).

T cell activation induces IL-2 and CD25 signaling, promoting IL-2-induced glycolysis that is characterized by the activation of mTOR and the upregulation of Glut1 (Finlay et al., 2012; Ray et al., 2015). The increase in glycolysis allows cells to generate the synthetic molecules needed for rapid proliferation and proper effector function. Proliferative effector T cells are highly sensitive to various forms of cell death, including Fas stimulation and cytokine withdrawal (Alderson et al., 1995; Snow et al., 2010; Larsen et al., 2017). The cytokine IL-15 is also important in proliferation. By contrast, IL-15 reduces glycolysis and promotes oxidative phosphorylation and T cell survival to the memory stage, although the mechanism of survival is not clear (van der Windt et al., 2012; Saligrama et al., 2014).

In addition to the critical role of metabolism in T cell activation and proliferation, the metabolic state of T cells may greatly influence their susceptibility to cell death. Given that caspases are frequently the mediators of cell death, we considered that metabolism might regulate the activity of certain caspases, and as such, set a level of susceptibility to cell death. We have previously observed that IL-2 selectively promotes caspase-3 activity whereas IL-15 inhibits its activation. Knowing that IL-15 promotes activity of complex I of the electron transport chain (ETC) and oxidative phosphorylation (van der Windt et al., 2012; Secinaro et al., 2018), we considered that other mechanisms of reducing glycolysis and enhancing complex I activity might also reduce caspase-3 activity.

Methylation-controlled J protein (MCJ) was recently identified as a negative regulator of complex I (Hatle et al., 2013). MCJ is a member of the DNAJ family of proteins, encoded by the gene *dnajc15* (Shridhar et al., 2001; Hatle et al., 2007, 2013). MCJ is located at the inner mitochondrial membrane and interacts with complex I of the ETC (Hatle et al., 2013). This interaction decreases complex I activity and reduces supercomplex formation of members of the ETC, which results in a decrease in mitochondrial respiration (Champagne et al., 2016). MCJ-deficient T cells thus manifest increased complex I activity, mitochondrial respiration, and provide more effective memory than wild-type T cells (Champagne et al., 2016). We therefore considered that regulation of MCJ expression may be a component of the linkage between metabolism and cell death.

Here, we observe that as T cells enter glycolysis via IL-2 *in vitro* to become effector T cells they strongly upregulate MCJ. Paralleling this was an increase of caspase-3 activity. Similar findings were observed *in vivo* with rapidly proliferating glycolytic CD4^-^CD8^-^ T cells from Fas-deficient *lpr* mice. By contrast, in MCJ-deficient IL-2 effector T cells caspase-3 activity was decreased. IL-15-cultured T cells downregulated MCJ expression through its gene methylation, which also paralleled reduced caspase-3 activity. These findings establish a close relationship between glycolysis, MCJ, and mitochondrial respiration, with a level of caspase-3 activity that is independent of Fas engagement.

## Results

### Induction of Glycolysis by IL-2 Increases Expression of MCJ and Reduced Complex I Activity Which Is Reversed by IL-15

**FIGURE 1:**
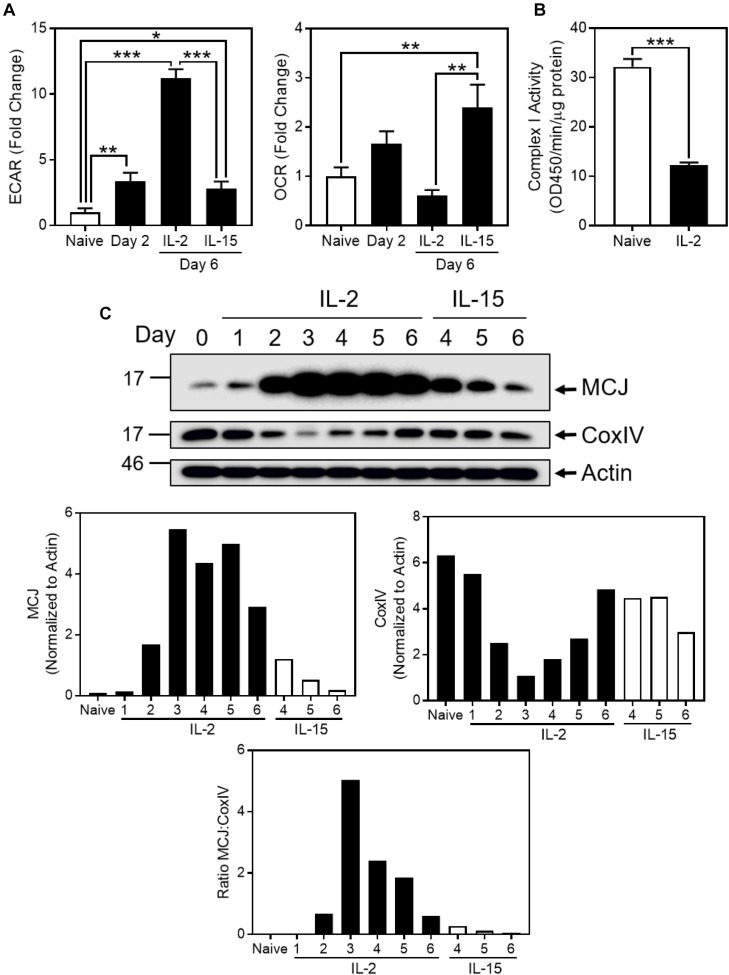
MCJ expression is upregulated with the induction of glucose utilization in CD8^+^ T cells. **(A–C)** CD8^+^ T cells were activated for 2 days with adherent anti-CD3/CD28, removed and cultured for a third day in IL-2, then washed and cultured for three additional days in either IL-2 or IL-15. **(A)** Relative baseline extracellular acidification rate (ECAR) and oxygen consumption rate (OCR) of CD8^+^ T cells, measured by extracellular flux analysis at days 0, 2, and 6 (one-way ANOVA with Tukey’s correction; mean ± SD; ^∗^*p* < 0.05; ^∗∗^*p* < 0.01; ^∗∗∗^*p* < 0.0001; *n* = 3 independent experiments). **(B)** Complex I activity in naïve or day 6 IL-2-cultured CD8^+^ T cells (*t*-test; mean ± SD; ^∗∗∗^*p* < 0.0001; *n* = 3 independent experiments). **(C)** Immunoblot and densitometry (normalized to actin) for methylation-controlled J (MCJ) protein, cytochrome c oxidase subunit IV (CoxIV), and actin in whole cell lysates of IL-2- or IL-15-cultured CD8^+^ T cells, day 0 through day 6 (blot and graphs are representative of two independent experiments). Graph of the MCJ:CoxIV ratio (representative of two independent experiments).

We modeled *in vitro* the metabolic switch that occurs in CD8^+^ T cells during the transition from naïve to effector and then to memory T cells by analyzing freshly purified CD8^+^ T cells before, and at various times after, activation with anti-CD3/CD28. After 2 days, cells were removed from the activation stimuli and cultured for an additional day in IL-2, then washed and recultured for an additional 3 days in cytokines known to induce differing metabolic states; IL-2 to induce glycolysis and effector T cells versus IL-15 to induce oxidative phosphorylation and memory T cells (van der Windt et al., 2012). IL-2-cultured activated CD8^+^ T cells manifested a rapid increase in glycolysis, as measured by extracellular acidification rate (ECAR) and a low oxygen consumption rate (OCR) on day 6, whereas IL-15-cultured CD8^+^ T cells had the opposite metabolic profile, consistent with oxidative phosphorylation, in agreement with previous reports (van der Windt et al., 2012; Figure 1A).

As oxygen consumption is primarily due to mitochondrial activity of the ETC, we examined complex I activity in naïve versus IL-2-cultured CD8^+^ T cells. Corresponding to the decrease in OCR as naïve T cells became glycolytic effector T cells with IL-2 stimulation, we observed a decrease in complex I activity in IL-2 CD8^+^ T cells (Figure 1B). We further investigated the possibility that the metabolic switch between oxidative phosphorylation and glycolysis reflected changes in MCJ, which physically associates with complex I and inhibits its activity (Hatle et al., 2013). MCJ expression in naïve CD8^+^T cells increased following activation with IL-2, peaking at days 3–5. In contrast, MCJ expression decreased rapidly in IL-15-cultured CD8^+^ T cells compared to IL-2, resembling resting naïve CD8^+^T cells (Figure 1C). Of further interest was that paralleling the increase in MCJ expression with IL-2, CoxIV (a subunit of complex IV) decreased, reaching low expression levels at days 3–4, and then increasing toward initial levels. This shows that the increase in MCJ was not the result of a global increase in mitochondrial mass, as reflected in the MCJ:CoxIV ratio (Figure 1C). IL-15-cultured-CD8^+^ T cells manifested the opposite profile with persistently high CoxIV, compared to their low levels of MCJ (Figure 1C).

### Inhibition of DNA Methyltransferases Results in the Expression of MCJ in IL-15-Cultured CD8^+^ T Cells

**FIGURE 2:**
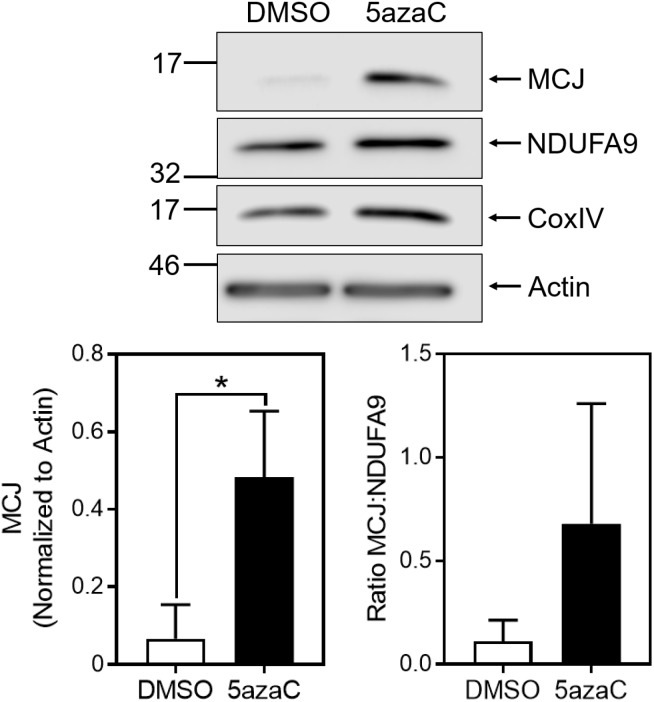
Inhibition of DNA methylation increases MCJ expression in IL-15-cultured CD8^+^ T cells. Anti-CD3/CD28-activated CD8^+^ T cells were cultured in IL-15 supplemented with 1 μM 5-azacytidine (5azaC) or an equivalent volume of DMSO for 48 h. Whole cell lysates were immunoblotted for MCJ, NDUFA9, CoxIV, and actin. Bar graph represents densitometry quantification of MCJ normalized to actin and the MCJ:NDUFA9 ratio (*t*-test; mean ± SD; ^∗^*p* < 0.05; MCJ:NDUFA9 ratio *p* = 0.1707; *n* = 3 independent experiments; blot is representative of three independent experiments).

Given the differences in MCJ expression between glycolytic IL-2-cultured cells and non-glycolytic IL-15-cultured cells, we investigated the possibilities that IL-15 reduces MCJ expression or glycolysis drives MCJ expression. The gene encoding MCJ, *dnajc15*, is known to be regulated by methylation-induced suppression (Shridhar et al., 2001; Strathdee et al., 2004). To investigate whether the reduction in MCJ by IL-15 was related to gene methylation we cultured activated CD8^+^ T cells in IL-15 with 1 μM 5-azacytidine (5azaC), a DNA methyltransferase inhibitor, or with an equivalent concentration of DMSO as a vehicle control for 2 days starting at day 3. We observed that 5azaC treatment resulted in a substantially increased expression of MCJ protein, and this was to a much greater extent than for complex I component NDUFA9, or complex IV component CoxIV (Figure 2). This was reflected in the MCJ:NDUFA9 ratio, indicating 5azaC treatment did not globally increase mitochondrial proteins (Figure 2).

### Glucose Utilization Increases MCJ Protein Expression

**FIGURE 3:**
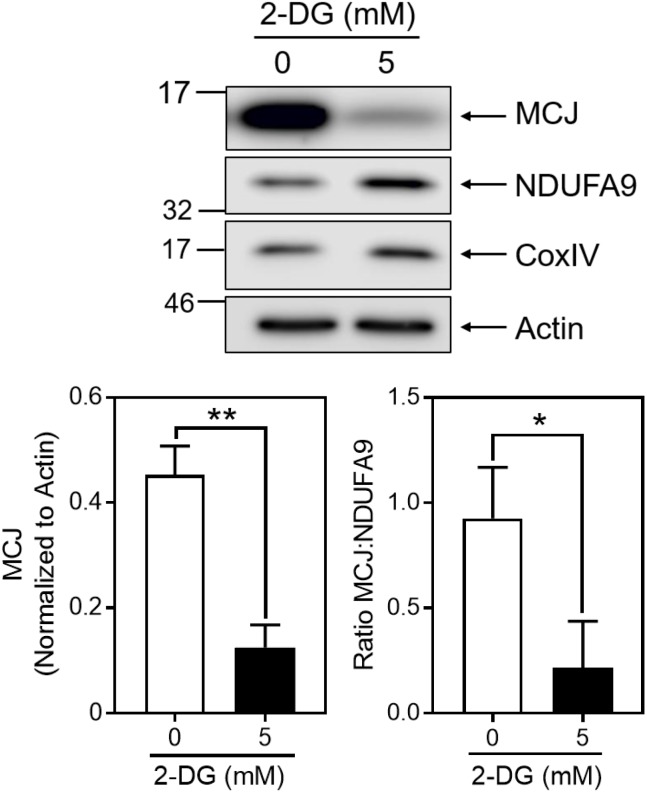
Inhibition of glycolysis decreases MCJ expression. Anti-CD3/CD28 activated CD8^+^ T cells were cultured in IL-2 ± 5 mM 2-deoxyglucose for 3 days. Whole cell lysates were immunoblotted for MCJ, NDUFA9, CoxIV, and actin. Bar graphs represent the densitometry quantification of MCJ normalized to actin, and the MCJ:NDUFA9 ratio (*t*-test; mean ± SD; ^∗^*p* < 0.05; ^∗∗^*p* < 0.01; *n* = 3 independent experiments; blot is representative of three independent experiments).

We directly examined the influence of glycolysis on MCJ expression using 2-deoxy-D-glucose (2-DG), which inhibits glycolysis after its phosphorylation by hexokinase (Wick et al., 1957). We cultured activated CD8^+^ T cells in IL-2 for 3 days in the absence or presence of 5 mM 2-DG, starting at day 3. Cells cultured with IL-2 plus 2-DG expressed dramatically less MCJ compared to IL-2 alone (Figure 3). Such decreases were not observed for NDUFA9 or CoxIV, as reflected in the MCJ:NDUFA9 ratio (Figure 3). These data demonstrate a link between MCJ expression and glucose utilization in CD8^+^ T cells.

### Reduced Glycolysis and Caspase-3 Activity of T Cells From MCJ-Deficient Mice

**FIGURE 4:**
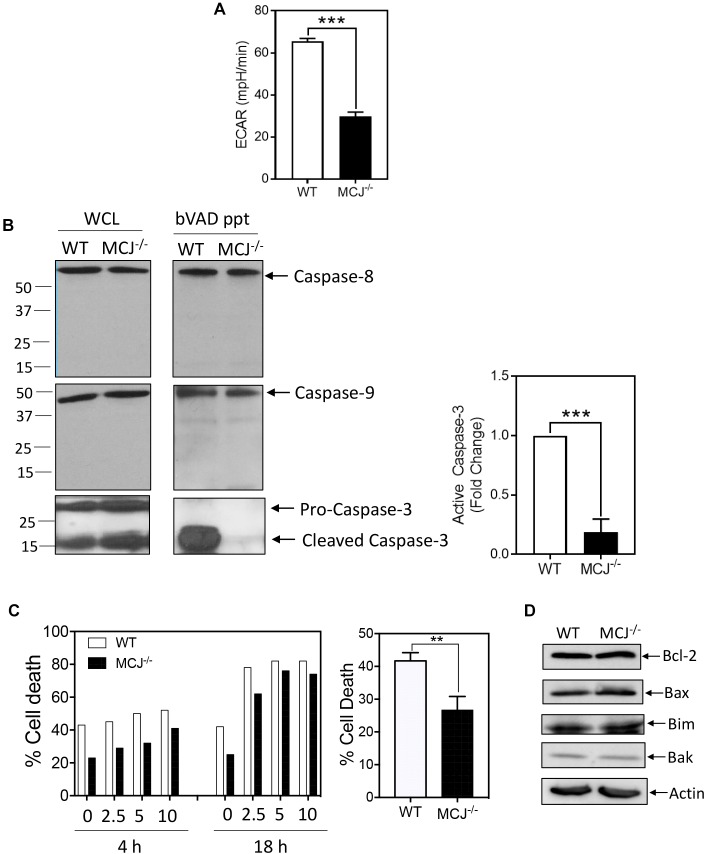
MCJ deficiency reduces glycolysis and caspase-3 activity CD8^+^ T cells. Day 2 anti-CD3/CD28-activated wild-type (WT) or MCJ-deficient (MCJ^-/-^) CD8^+^ T cells were cultured in IL-2 for an additional **(A)** 2 or **(B–D)** 4 days. **(A)** ECAR was measured by extracellular flux analysis. Bar graph is a summary of five samples in one experiment (*t*-test; mean ± SD; ^∗∗∗^*p* < 0.0001; representative of two independent experiments). **(B)** Active caspases were precipitated from WT or MCJ^-/-^ whole cell lysates using biotin-VAD (bVAD ppt). Whole cell lysates (WCL) and bVAD precipitates were immunoblotted for caspase-8, caspase-9, and caspase-3. Bar graph indicates fold change in active caspase-3 compared to WT (*t*-test; mean ± SD; ^∗∗∗^*p* < 0.0001; *n* = 3 independent experiments). **(C)** On day 6 WT or MCJ^-/-^ cells were restimulated with the indicated doses of anti-CD3 for 4 or 18 h in one experiment, or with anti-CD3 at 10 μg/mL for 4 h in three experiments. Cell death was measured by Live/Dead staining and flow cytometry (*t*-test; mean ± SD; ^∗∗^*p* < 0.005; *n* = 3 independent experiments). **(D)** Whole cell lysates were immunoblotted for Bcl-2, Bax, Bim, and Bak (*n* = 3 independent experiments).

Given the link between glucose utilization and MCJ expression, we measured glycolysis in IL-2-cultured effector CD8^+^ T cells derived from either MCJ-deficient (MCJ^-/-^) mice or wild-type control mice by extracellular flux analysis at day 4. The MCJ^-/-^ T cells were less glycolytic than the WT T cells (Figure 4A). We have previously reported that glycolysis drives caspase-3 activity (Secinaro et al., 2018). Since MCJ^-/-^ T cells were less glycolytic than wild-type T cells, we hypothesized the MCJ^-/-^ T cells would have reduced caspase-3 activity. Cell lysates were prepared in the presence of biotin-VAD (bVAD), which labels active caspases (Zhou et al., 2006). Active caspases were then precipitated from lysates with avidin-sepharose, and precipitates immunoblotted for caspases-3, -8, and -9. We observed that MCJ^-/-^ CD8^+^ T cells manifested a pronounced decrease in active caspase-3 but no detectable differences in the activities of caspase-8 or caspase-9 (Figure 4B). CD8^+^ T cells deficient in MCJ have been shown previously to be resistant to cell death during a simulated immune contraction phase using cytokine withdrawal (Champagne et al., 2016). However, the mechanism for this increased survival is not known. Since cell death by either cytokine withdrawal or T cell receptor restimulation is mediated by caspases (Snow et al., 2010), we examined whether this resistance to cell death extended also to restimulation-induced cell death (RICD). We restimulated day 6 effector wild-type or MCJ^-/-^ T cells with varying doses of anti-CD3 for 4 or 18 h, and measured cell death by Live/Dead stain and flow cytometry. Consistent with our previous observations, wild-type effector T cells cultured in IL-2 manifested moderate levels of spontaneous death prior to anti-CD3 restimulation, and this was reduced in MCJ^-/-^ T cells (Figure 4C). These differences persisted following anti-CD3 restimulation. This difference was not due to detectable differences in the expression levels of Bcl-2, Bax, Bim, or Bak (Figure 4D).

### Highly Proliferative and Glycolytic T Cells *in vivo* Manifest High Levels of MCJ and Increased Active Caspase-3

**FIGURE 5:**
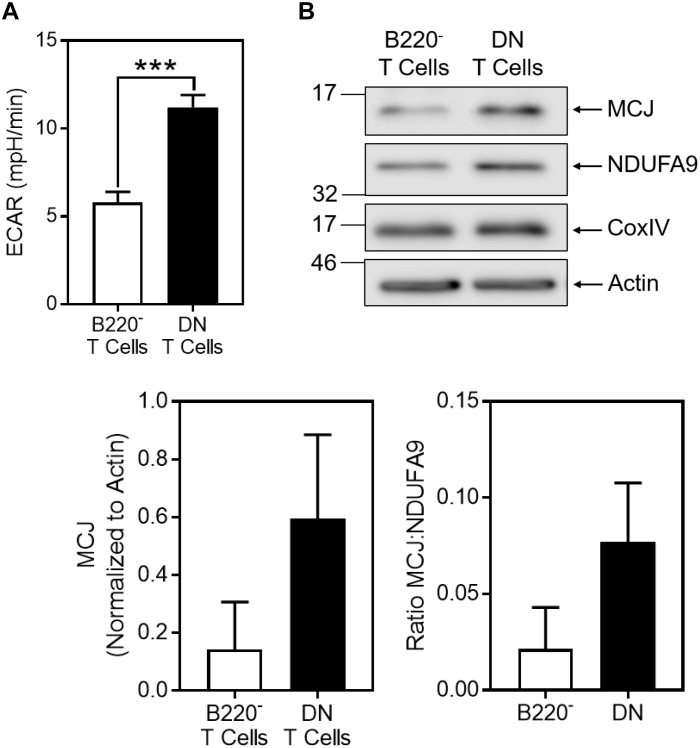
MCJ expression is increased *in vivo* in highly glycolytic CD4^-^CD8^-^ T cells from *lpr* mice. Freshly isolated CD4^-^CD8^-^ double negative (DN) T cells or CD4^+^ + CD8^+^ (B220^-^) T cells from the same *lpr* mice were purified from lymph nodes by negative selection. **(A)** ECAR was measured by extracellular flux analysis. Bar graph is a summary of five samples in one experiment (*t*-test; mean ± SD; ^∗∗∗^*p* < 0.0001; representative of two independent experiments). **(B)** Whole cell lysates were immunoblotted for MCJ, NDUFA9, CoxIV, and actin. Bar graphs represent the densitometry quantification of MCJ normalized to actin, and the MCJ:NDUFA9 ratio (mean ± SD; *n* = 2 independent experiments).

To examine whether these observations *in vitro* extend to proliferative glycolytic T cells *in vivo*, we examined a highly proliferative T cell subset that exists in Fas-deficient *lpr* mice. CD4^-^CD8^-^ (double negative, DN) T cells accumulate in *lpr* mice with age and our previous studies have shown that by *in vivo* BrdU labeling a remarkable 18% of this subset undergoes cell cycling in a 24 h period compared with only about 5–6% of the CD4^+^ and CD8^+^ (B220^-^) T cells from the same mice (Fortner et al., 2017). Consistent with this, we observed that freshly isolated *lpr* DN T cells were highly glycolytic, as revealed by ECAR, compared to the B220^-^ T cells (Figure 5A). In parallel with their high glycolytic state, the DN T cells also expressed high levels of MCJ compared to the B220^-^ T cell subset (Figure 5B). Similar increases in NDUFA9 or CoxIV were not observed in the DN population, as reflected in the MCJ:NDUFA9 ratio (Figure 5B).

**FIGURE 6:**
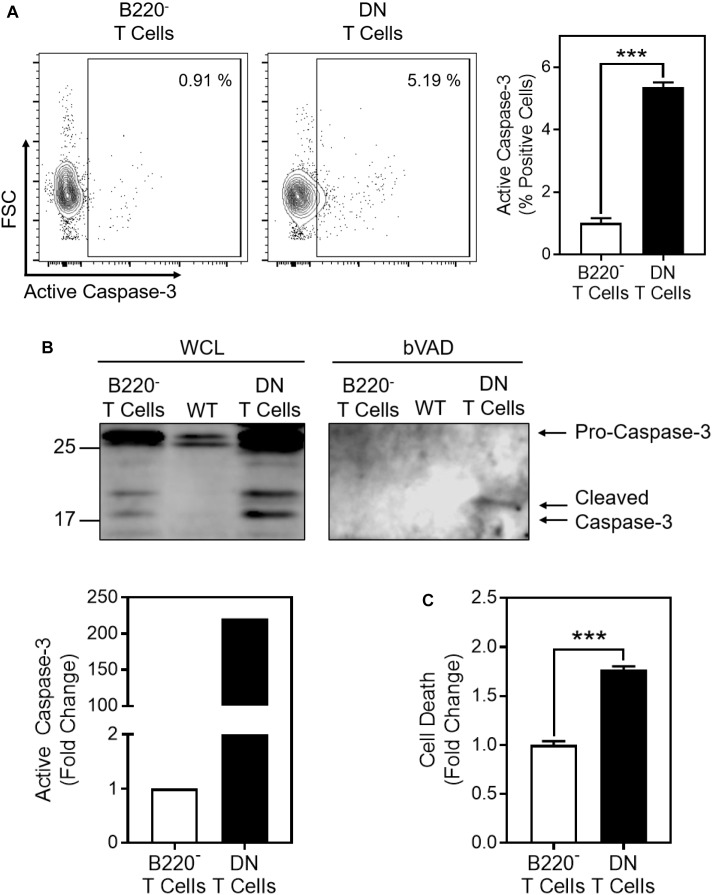
Cell death and active caspase-3 are increased in CD4^-^CD8^-^ T cells from *lpr* mice. Freshly isolated CD4^-^CD8^-^ DN T cells or CD4^+^ + CD8^+^ (B220^-^) T cells from the same *lpr* mice were purified from lymph nodes by negative selection. **(A)** Representative contour plot of flow cytogram for active caspase-3. The box indicates the gate for active caspase-3 based on isotype control staining. Number inserts indicate the percent active caspase-3-positive cells. Bar graph is a summary of quantification of percent active caspase-3-positive cells from three samples in one experiment (*t*-test; mean ± SD; ^∗∗∗^*p* < 0.0001; plots and bar graph are representative of two independent experiments). **(B)** bVAD precipitation of active caspases from DN T cells, B220^-^ T cells, and wild-type lymphocytes (WT), immunoblotted for caspase-3. Graph is of the relative densitometry of active caspase-3. **(C)** Cell death was measured by Live/Dead staining and flow cytometry in DN or B220^-^ T cells after overnight incubation at 37°C (*t*-test; mean ± SD; ^∗∗∗^*p* < 0.0001; bar graph depicts three samples from one experiment and is representative of two independent experiments).

Given the increased glycolysis and MCJ expression in the DN T cells, we examined whether there was also an increase in caspase-3 activity. We first measured caspase-3 activity by flow cytometry and found a five-fold increase in cells positive for active caspase-3 in the DN T cells compared to the B220^-^ T cells (Figure 6A). This was confirmed by bVAD precipitation (Figure 6B). This paralleled an almost two-fold increase in spontaneous cell death in the DN T cells compared to B220^-^ T cells (Figure 6C).

## Discussion

The current findings highlight the coordinated regulation between induction of glycolysis and the negative regulation of mitochondrial respiration and oxygen consumption in the ETC in T cells. We show as resting naïve CD8^+^ T cells are activated and increase glycolysis in the presence of IL-2, they decrease electron transport and oxygen consumption by reducing expression of the ETC complexes. Congruently, expression of the complex I negative regulator MCJ is increased, further reducing electron transport and oxidative phosphorylation (OXPHOS). The presence of MCJ also acts synergistically with glycolysis to promote caspase-3 activation. This likely acts as a safeguard to prevent the accumulation of highly proliferative CD8^+^ T cells that might cause damage to self-tissues.

The metabolic switch from OXPHOS in naïve and memory T cells to glycolysis in effector T cells is crucial for supporting their rapid proliferation and synthetic capacity. Under such proliferative conditions, it is important to shunt glucose metabolites into synthetic pathways, such as the pentose phosphate pathway, rather than the Krebs cycle and ATP production by the ETC. It may thus be beneficial in certain instances for glycolytic T cells to actively suppress the ETC by reducing expression of its components as well as upregulating an inhibitor, MCJ. This notion is consistent with the observation that inhibition of glycolysis with 2-DG decreased the MCJ:NDUFA9 ratio.

The expansion of proliferative effector T cells is paralleled by an increase in caspase-3 activity, which sensitizes them to RICD. We have previously shown that caspase-3 activity is regulated in part by the glycolytic state of the cell, as caspase-3 activity in IL-2 effector T cells is reduced in the presence of 2-DG (Secinaro et al., 2018). The current observation that IL-2 induces MCJ expression and reduced mitochondrial respiration would require T cells to upregulate glycolysis to supply needed ATP. Conversely, activated CD8^+^ T cells deficient in MCJ manifest increased mitochondrial respiration and oxygen consumption as well as compensatory reduced glycolysis compared to wild-type CD8^+^ T cells. The reduced glycolytic activity would serve to decrease the activation of caspase-3 and sensitivity to reactivation-induced cell death. This may help explain the increased memory response of MCJ^-/-^ CD8^+^ T cells to influenza infection (Champagne et al., 2016). These findings closely resemble IL-15-cultured T cells, which also have reduced MCJ expression, increased oxygen consumption and reduced active caspase-3 compared to IL-2-cultured T cells (van der Windt et al., 2012; Saligrama et al., 2014; Secinaro et al., 2018). As both IL-15 and MCJ deficiency contribute to increased T cell memory, their common reduction in active caspase-3 is likely a significant contributing factor in enhancing their survival.

The *lpr* CD4^-^CD8^-^ (DN) T cells provided a useful *in vivo* source of rapidly proliferating T cells in which to test our *in vitro* observations. We have previously shown using *in vivo* BrdU labeling of proliferating cells that a remarkable 18% of the DN T cells in *lpr* mice divide within a given 24 h period (Fortner et al., 2017). This extraordinary rate of proliferation was paralleled by a high rate of glycolysis as well as elevated levels of MCJ. The high levels of caspase-3 activity in *lpr* DN T cells paralleled their rapid cell death *ex vivo*, which was obviously independent of Fas expression since Fas is absent on *lpr* T cells. This serves to underscore that caspase-3 activity in highly proliferative T cells is likely independent of death receptor ligation. This is also consistent with our previous observations that whereas caspase-3 activity is higher in IL-2-cultured T cells than IL-15-cultured T cells, caspase-8 activity is quite similar, or even slightly higher, in IL-15-cultured T cells (Saligrama et al., 2014). Similarly, in the current studies MCJ-deficient IL-2-cultured T cells manifested as much or more active caspase-8 than wild-type IL-2-cultured T cells, despite MCJ-deficient T cells having greatly reduced active caspase-3. Thus, caspase-3 activity in proliferating T cells is independent of active upstream caspase-8.

The elevated expression of MCJ in glycolytic T cells may serve a variety of critical functions. By inhibiting complex I and electron transport, it may reduce ROS generation, which could reduce ROS-mediated inactivation of caspase-3 (Mitchell and Marletta, 2005; Huang et al., 2008; Saligrama et al., 2014; Secinaro et al., 2018) and promote susceptibility to cell death of rapidly proliferating T cells. Increased MCJ could thus help divert carbon fuel sources away from mitochondrial respiration and toward anabolic pathways, as is seen in actively proliferating IL-2-cultured T cells.

The current findings establish a close linkage between active T cell proliferation, glucose utilization, and activation of caspase-3. That glycolysis promotes activation of selectively caspase-3, whereas oxidative phosphorylation actively inhibits caspase-3 activity through glutathionylation and S-nitrosylation, underscores the tight regulation of caspase-3 activity by the metabolic state of T cells. Such a linkage may represent an adaptation to ensure that proliferating glycolytic T cells do not escape regulation that might promote tumor progression or an autoimmune diathesis. Thus, inhibitors of MCJ, which are currently under development, might considerably augment the survival of T cells during vaccination or immunotherapy.

## Materials and Methods

### Mice

C57BL/6J mice (Jackson Laboratory, Bar Harbor, ME, United States), MRL/MpJ-Fas^lpr^/J mice (Jackson Laboratory), MCJ^-/-^ mice (Hatle et al., 2013), were housed in an American Association for Accreditation of Laboratory Animal Care (AAALAC)-approved animal facility at the University of Vermont Larner College of Medicine. Tissues from MCJ^-/-^ and C57BL/6J mice were harvested at 2–6 months of age and tissues from MRL/MpJ-Fas^lpr^/J mice were harvested at 4 months of age. Protocols were approved by the Institutional Animal Care and Use Committee.

### Cell Purification

CD8^+^ T cells were purified by negative selection from mouse lymph nodes (axillary, inguinal, brachial, and cervical) and spleens as described previously (Saligrama et al., 2014). In brief, tissues were homogenized through nylon mesh and Gey’s solution was used to lyse red blood cells. Combined splenocytes and lymph node cells were incubated for 30 min on ice with the following antibodies: anti-MHC class II (M5/114/15/2), anti-B220 (RA3-6B2), anti-CD11b (M1/70), and anti-CD4 (GK1.5). Cells were then rocked with goat anti-rat coated magnetic beads (Qiagen, Germantown, MD, United States) at a 10:1 bead:cell ratio for 45 min at 4°C. Beads and bound cells were removed with a magnet.

CD4^-^CD8^-^ B220^+^ DN and B220^-^ (CD4^+^ + CD8^+^) T cells were purified from the lymph nodes of MRL/MpJ-Fas^lpr^/J in the same manner with the following antibodies: DN T cells; anti-MHC class II (M5/114/15/2), anti-CD11b (M1/70), anti-CD4 (GK1.5), anti-CD8 (Tib105), and anti-kappa (187.1). B220^-^ T cells; anti-MHC class II (M5/114/15/2), anti-CD11b (M1/70), and anti-B220 (RA3-6B2). Wild-type lymphocytes were obtained from C57BL/6J mouse lymph nodes.

### Cell Culture

Naïve CD8^+^ T cells were cultured in RPMI-1640 (Corning, Manassas, VA, United States), supplemented with 25 mM HEPES, 100 U/mL penicillin-streptomycin (Thermo Fisher Scientific, Waltham, MA, United States), 50 μM 2-mercaptoethanol, 5% bovine calf serum (GE Healthcare HyClone, Logan, UT, United States), 2.5 mg/L glucose, 1 mM pyruvate, 2 mM glutamine, and 10 μg/mL folate (RPMI-C), and stimulated on 10 μg/mL plate-bound anti-CD3 clone 145-2C11 (Bio X Cell, West Lebanon, NH, United States) and soluble anti-CD28 ascites clone 37–51 (1:1000), supplemented with 50 U/mL IL-2 (Cetus, Emeryville, CA, United States) at 37°C and 5% CO_2_ for 2 days. The activated CD8^+^ T cells were then removed from stimulation and cultured in RPMI-C and 50 U/mL IL-2 for an additional day. For studies comparing IL-2- to IL-15-cultured CD8^+^ T cells, the cells were then washed 3 times to remove cytokines and cultured in RPMI-C supplemented with either 50 U/mL IL-2 or 20 ng/mL IL-15 (a kind gift from Amgen, Thousand Oaks, CA, United States) for 3 days or the number of days indicated. For studies of the inhibition of glycolysis, cells were cultured in RPMI-C medium with 50 U/mL IL-2 with or without 5 mM 2-DG (Sigma-Aldrich, St. Louis, MO, United States) for 3 days. For studies inhibiting DNA methylation, cells were washed three times to remove cytokines and cultured in RPMI-C supplemented with 20 ng/mL IL-15 and 1 μM 5azac (Sigma-Aldrich) or an equal concentration of DMSO for 2 days.

### Biotin-VAD Precipitation of Active Caspases

Cells were washed once with PBS/1% BSA, washed again with PBS, then lysed for 30 min on ice in Lysis Buffer supplemented with 20 μM bVAD-fmk (MP Biomedicals, Solon, OH, United States). Protein was quantified by Bradford assay. 400–600 μg of protein in 300 μL of Lysis Buffer was rocked with 40 μL of Sepharose 4B beads (Sigma-Aldrich) at 4°C for 2 h. The beads were removed and the supernatants were rocked with 60 μL streptavidin-Sepharose beads (Thermo Fisher Scientific) at 4°C overnight. The beads bound with active caspases were washed 3 times with Lysis Buffer without protease inhibitor, then boiled for 5 min in Laemmli loading buffer supplemented with 2-ME and analyzed by immunoblot for active caspases as described below.

### Immunoblot Assays

Cells were washed in PBS containing 1% bovine serum albumin (PBS/1% BSA), washed again with PBS, then lysed for 30 min on ice in Lysis Buffer [0.5% Nonidet P-40, 20 mM Tris–HCl (pH 7.4), 150 mM NaCl, 2 mM sodium orthovanadate, 10% glycerol, and Complete Protease Inhibitor (Roche Diagnostics, Indianapolis, IN, United States)]. Protein concentration of each lysate was determined by Bradford Assay (Bio-Rad, Hercules, CA, United States). Lysates were boiled in Laemmli loading buffer supplemented with 2-mercaptoethanol (2-ME) for 5 min. Proteins were separated by SDS–PAGE on a 15% acrylamide gel and transferred to a polyvinylidene difluoride (PVDF) membrane (Bio-Rad). Membranes were blocked in 4% milk in Tris-buffered saline with 0.1% Tween-20 (American Bioanalytic, Natick, MA, United States) at room temperature for 1 h. The following antibodies were used for protein detection: anti-caspase-3, 585 rabbit polyclonal antibody (a kind gift from Dr. Yuri Lazebnik, Cold Spring Harbor Laboratories, Cold Spring Harbor, NY, United States), anti-caspase-8 (a kind gift from Dr. Andreas Strasser, The Walter and Eliza Hall Institute of Medical Research, Melbourne, Australia), anti-MCJ (Hatle et al., 2007), anti-CoxIV (Cell Signaling, Danvers, MA, United States), anti-NDUFA9 (Abcam, Cambridge, United Kingdom), anti-β-actin (Sigma-Aldrich), anti-mouse IgG HRP, anti-rabbit IgG HRP, and anti-rat IgG HRP (all from Jackson Laboratory). Densitometry was performed using ImageQuant v8.1 software (GE Healthcare, Chicago, IL, United States).

### Metabolic Analysis

Extracellular acidification rates and OCR were measured with the Seahorse XFe 96 Analyzer (Agilent Technologies, Santa Clara, CA, United States) according to the manufacturer’s specifications. Analysis was performed with the Wave Software v2.4 or v2.6 (Agilent Technologies).

### Flow Cytometry of Active Caspase-3

Cells were washed in PBS and stained in PBS with Live/Dead Fixable Blue Dead Cell Stain (Thermo Fisher Scientific) on ice for 25 min. Cells were washed with PBS/1% BSA and fixed with 2% formaldehyde (v/v) on ice for 15 min. The fixed cells were then washed with PBS/1% BSA and permeabilized with PBS/1% BSA supplemented with 0.03% saponin (PBS/1% BSA/0.03% saponin) on ice for 10 min. Fixed and permeabilized cells were then washed and incubated with anti-cleaved caspase-3 Alexa 647 (Cell Signaling) in PBS/1% BSA/0.03% saponin on ice for 30 min. Cells were washed, fixed in 1% formaldehyde (v/v), and analyzed on an LSRII (BD Biosciences).

### Cell Death

Restimulation-induced cell death was induced in day 6 wild-type (WT) or MCJ^-/-^ CD8^+^ T cells by incubation on plate-bound anti-CD3 (2.5–10 μg/mL) for 4 or 18 h at 37°C. Cells were removed from the plate and stained with Live/Dead Fixable Blue Dead Cell Stain (Thermo Fisher Scientific), fixed in 1% formaldehyde (v/v), and analyzed by flow cytometry.

### Complex I Activity Measurement

Cells were washed twice with PBS then resuspended in PBS and assayed for complex I activity using the Complex I Enzyme Activity Microplate Kit (MitoSciences, Eugene, OR, United States) according to the manufacturer’s specifications. 40–100 μg of protein was used for each sample. Complex I rates were calculated per microgram of protein used in the assay.

### Statistical Analysis

Statistical analyses were performed using the graphing software Prism v7 (GraphPad Software, La Jolla, CA, United States). The following statistical tests were used: paired and unpaired *t*-test when comparing two conditions (e.g., DMSO compared to treatment), one-way ANOVA with Tukey’s test for correction for multiple comparisons when comparing multiple conditions (e.g., naïve compared to Day 2 compared to Day 6) and two-way ANOVA with Sidak test for correction for multiple comparisons when comparing multiple variables across multiple conditions (e.g., wild-type versus MCJ^-/-^ with and without anti-CD3 restimulation). All data met the assumptions of the statistical tests used and variation among the compared groups was similar.

## Data Availability

The datasets generated for this study are available on request to the corresponding author.

## Author Contributions

MS, KF, MR, and RB designed the experiments presented in this manuscript, which were performed by MS, KF, CC, and RB. MS and RB analyzed the resulting data. MS and RB wrote the manuscript, which was edited by MS, KF, MR, and RB.

## Conflict of Interest Statement

The authors declare that the research was conducted in the absence of any commercial or financial relationships that could be construed as a potential conflict of interest.
